# Signal pathways in astrocytes activated by cross-talk between of astrocytes and mast cells through CD40-CD40L

**DOI:** 10.1186/1742-2094-8-25

**Published:** 2011-03-16

**Authors:** Dae Yong Kim, Gwan Ui Hong, Jai Youl Ro

**Affiliations:** 1 Department of Pharmacology and Samsung Biomedical Research Institute, Sungkyunkwan University School of Medicine, 300 chunchun-dong, Jangan-ku, Suwon, 440-746, South Korea

## Abstract

**Background:**

Astrocytes, which play an active role in chronic inflammatory diseases like multiple sclerosis, exist close to mast cells with which they share perivascular localization. We previously demonstrated the possibility that astrocytes and mast cells interact in vitro and in vivo. This study aimed to investigate the signaling pathways and the role for astrocytes in the interaction of astrocytes and mast cells.

**Methods:**

We co-cultured human U87 glioblastoma (U87) and human mast cell-1 (HMC-1) cell lines, and mouse cerebral cortices-derived astrocytes and mouse bone marrow-derived mast cells (BMMCs). Intracellular Ca^2+ ^([Ca^2+^]_i_) was measured by confocal microscopy; CD40 siRNA by Silencer Express Kit; small GTPases by GTP-pull down assay; PKCs, MAPKs, CD40, CD40L, Jak1/2, STAT1, TNF receptor 1 (TNFR1) by Western blot; NF-κB and AP-1 by EMSA; cytokines by RT-PCR. An experimental allergic encephalomyelitis (EAE) model was induced using myelin oligodendrocyte glycoprotein (MOG) peptide and pertussis toxin in mice. Co-localization of TNFR1 and astrocytes in EAE brain tissues was determined by immunohistochemistry.

**Results:**

Each astrocyte co-culture had increases in [Ca^2+^]_i _levels, release of cytokines and chemokines; activities of Rho-family GTPases, NF-κB/AP-1/STAT1^727^, and Jack1/2, STAT1^701^. These effects were inhibited by anti-CD40 antibody or CD40 siRNA, and signaling pathways for Jak1/2 were inhibited by anti-TNFR1 antibody. EAE score, expression of TNFR1, and co-localization of TNFR1 and astrocytes were enhanced in brain of the EAE model. Anti-CD40 antibody or 8-oxo-dG pretreatment reduced these effects in EAE model.

**Conclusions:**

These data suggest that astrocytes activated by the CD40-CD40L interaction in co-culture induce inflammatory cytokine production via small GTPases, and the secreted cytokines re-activate astrocytes via Jak/STAT1^701 ^pathways, and then release more cytokines that contribute to exacerbating the development of EAE. These findings imply that the pro-inflammatory mediators produced by cell-to-cell cross-talk via interaction of CD40-CD40L may be as a promising therapeutic target for neurodegenerative diseases like MS.

## Background

Astrocytes, which are known as a major glial cell type, have important physiological properties in central nerve system (CNS) homeostasis. Astrocytes have a dynamic role in regulating neuronal function [1], and play an active and dual role in CNS inflammatory diseases such as multiple sclerosis (MS) [2].

MS is a progressive and neurodegenerative disease of the CNS. A major pathological hallmark of MS is the presence of demyelinated lesions [3,4]. In the active phase of this disease, which is known to be caused in the recruitment and activation of various cell types such as T cells [5], macrophages and dendritic cells [6] etc., mast cells [6,7] and astrocytes [8] have been reported as an effector cells, although these cells remain to be further determined. An accumulation of mast cells in MS plaques and normal appearing white matter observed by histopathological analysis [9,10], an elevation of mast cell specific enzyme (tryptase) in the cerebrospinal fluid (CSF) of MS patients [11], and an increase of mast cell markers (FcεRI, tryptase and chymase) [12] show the implication of mast cells in the pathophysiology of MS. Moreover, Mast cells related to experimental allergic encephalomyelitis (EAE) in monkey [13,14] and mice [15-19] as an animal model of MS were previously reported by others and our laboratories. However, it has been reported that mast cells are dispensable for development of disease [20], although they accumulate in the brain and CNS [18,19,21] and the reconstitution of mast cell population in W/W(v) mice, which are deficient in c-kit receptor, restores induction of early and severe disease to wild-type levels [19].

Astrocytes participate in immune function through the specific loss of a cytokine receptor like gp130, or through reduction of nuclear factor-κB (NF-κB) signaling [22]. Astrocytes lead to chronic inflammation and progressive neurodegeneration by overexpression of several cytokines such as interleukin (IL)-1β, tumor necrosis factor (TNF)-α, interferon (IFN)-γ, IL-6, IL-12, and transforming growth factor (TGF)-β [23,24], and by overexpression of chemokine like CCL2 (MCP-1) [25]. The cytokine TNF-α is also an important factor in the regulation of neuronal apoptotic cell death. TNF-α mRNA expression in blood mononuclear cells is correlated with disease activity in relapsing-remitting MS [26], while high IL-6 levels in the CNS [27] and TNF-α release in astrocytes [28] are correlated with the development of EAE in rats. Thus, future challenges include determining how individual cytokines and chemokines produced by astrocytes influence the development of inflammation and the behavior of infiltrating immune cell populations.

In the CNS, the co-stimulatory molecule CD40 is expressed in a variety of cells including astrocytes and microglia, and the natural ligand of CD40 (CD40L) belongs to the TNFR superfamily [29]. Interaction of CD40 on astrocytes and CD40L on the infiltrating T cells and other resident CNS cells such as monocytic cells, natural killer cells and mast cells, trigger a series of intracellular signaling events that promote the production of a wide array of cytokines, chemokines and neurotoxins [30]. In the mouse [31] and monkey [32] EAE, treatment with anti-CD40 antibody prevented disease development and reduced clinical signs.

We previously demonstrated that mast cells co-cultured with astrocytes are activated by CD40-CD40L interaction, and the activated mast cells induce release of mediators that participate in pathophysiology of chronic neurodegenerative diseases like MS [18]. However, the role of astrocytes activated in the co-culture is not yet clarified. Therefore, we hypothesized that both cells are bi-directionally activated in vitro and in vivo, and examined the signaling pathways and role for astrocytes in the co-culture system and EAE model. We observed that cross-talk between astrocytes and mast cells through CD40-CD40L produces inflammatory cytokines by Rho-family GTPases, and the produced cytokines re-activate astrocytes through cytokine-receptor-Jak1/2 and STAT1 on tyrosine^701 ^signaling pathways.

## Methods

### Cell culture

U87 glioblastoma cell lines were obtained from Korea Cell Line Bank (Seoul, Korea) and grown in Dulbecco's modified Eagle's medium (DMEM) (Gibco, Carlsbad, CA) supplemented with 10% fetal bovine serum (FBS; Welgene, Daegu, Korea), 10 U/ml penicillin and 10 μg/ml streptomycin (Gibco, Carlsbad, CA) at 37°C in a 5% CO_2 _atmosphere.

HMC-1 cells (human mast cell line) were kindly provided by Dr. J. H. Butterfield (Mayo Clinic, Rochester, MN). Cells were cultured in Iscoves modified Dulbecco's medium (IMDM; Gibco, Carlsbad, CA) containing 10% FBS at 37°C in a 5% CO_2 _atmosphere. These culture conditions were designated as control medium.

### Preparation of primary brain astrocytes and bone marrow-derived mast cells (BMMCs)

Primary brain astrocytes were isolated from the cerebral cortices of 1 day-old BALB/c mice as previously described [18]. In brief, animals were sacrificed by decapitation, meninges were removed, and cortices were minced and gently dissociated in Hank's balanced salt solution (HBSS; Sigma-Aldrich, St. Louis, MO). Cells were supplemented with DMEM containing 5% FBS, transferred into 75 cm^2 ^culture flasks (5 × 10^10 ^cells/flask), and incubated at 37°C in a humidified atmosphere of 95% air, 5% CO_2_. After 14 days of culturing, floating microglia was removed by shaking the flask vigorously. More than 95% of cells were stained for astrocyte specific glial fibrillary acidic protein (GFAP; Sigma-Aldrich, St. Louis, MO).

Bone marrow cells were flushed from femurs and tibias of BALB/c mice (female, 8 weeks old) as described previously [18]. Briefly, red blood cells were lysed using 0.1 M NH_4_Cl, and the remaining cells were washed, resuspended, and cultured for 5 weeks in RPMI-1640 (Gibco, Carlsbad, CA) supplemented with 10% FBS and 50% WEHI-3B conditioned media which contained IL-3. BMMCs (5 × 10^4 ^cells) were collected onto object glasses by cytospin (400 × g, 3 min). Cells were fixed in methanol for 2-3 min, and then stained with May Grünwald solution for 15 min followed by Giemsa solution for 10 min and by washing with H_2_O, and then BMMCs were confirmed under microscope. Purity of BMMCs was more than 95% of total cells.

### Co-culture of astrocytes and mast cells

U87 cells or primary brain astrocytes (3 × 10^6 ^cells) were grown in 75 cm^2 ^flasks until confluent, and then HMC-1 cells or BMMC (1 × 10^6 ^cells), respectively, were added to each astrocyte flask because mast cells are floating cells. The cells were co-cultured for up to 24 h. *In vivo*, brain astrocytes outnumber mast cells, and we chose a 3:1 ratio of mast cells and astrocytes to activate astrocytes. After co-culture, mast cells were separated from astrocytes attached to the flask by gentle shaking. Astrocytes were separated from flasks using trypsin treatment and harvested by centrifugation (800 × g; referred to co-cultured-U87 cells or co-cultured-primary astrocytes). The optimal concentration and time for anti-CD40 antibody treatment, 8-oxo-dG pretreatment or anti-TNFR1 antibody treatment were 300 ng/ml for 1 h, 300 μg/ml for 10 min, 300 ng/ml for 30 min, respectively, obtained in preliminary experiments.

For inhibition experiments, U87 cells (1 × 10^6 ^cells) were pre-incubated for 1 h, and Jak inhibitor (10 μM AG490), PKC inhibitors (5 nM staurosporine and Gö6976), MAP kinase inhibitors (50 μM PD98059 for ERK, 10 μM SP600125 for JNK, and 10 μM SB203580 for p38) or Ca^2+ ^influx inhibitor 2-aminoethoxydiphenyl borate (100 μM 2-APB) were pretreated in astrocytes 5, 10 and 10 min, respectively, before initiating co-culture.

### Measurement of intracellular [Ca^2+^]_i _levels

Co-cultured-U87 cells or -primary astrocytes (1 × 10^6 ^cells) were seeded on cover slides, and each slide was then incubated for 30 min with Fluo-3 AM (5 μM) (Sigma-Aldrich, St. Louis, MO). The intracellular calcium ([Ca^2+^]_i_) levels in co-cultured-astrocytes were analyzed using LSM 510 laser scanning microscopy (Carl Zeiss, oberkochen, Germany) [18]. Intensity for [Ca^2+^]i level indicated the ratio of control intensity (intensity of control fluorescence = 1).

### Reverse transcriptase-polymerase chain reaction (RT-PCR)

Expression of cytokines or chemokines (IL-1β, IL-6, TNF-α, MCP-1, RANTES, and IP-10) were analyzed by RT-PCR. Total cellular RNA was isolated from the co-cultured-astrocytes (3 × 10^6 ^cells) using Trizol reagent (Molecular Research Center Inc., Cincinnati, OH). RT-PCR was performed in a final volume of 50 μl using a amfiRivert 1-step RT-PCR kit (GenDEPOT, Barker, TX) in an automated thermal cycler (BIOER Technology Co., Hangzhou, China). PCR assays were performed for 35 cycles. Each cycle consisted of the following steps: denaturation at 94°C for 30 seconds, annealing at 56°C for 45 seconds, and extension at 72°C for 1 min. PCR products were analyzed using a 1% agarose gel containing ethidium bromide (EtBr; Sigma-Aldrich, St. Louis, MO) [18].

The primer sequences used were as follows: human IL-1β sense, 5'-AAG GAG AAC CAA GCA ACG ACA AAA-3'; anti-sense, 5'-CCT GTA TGC CTC TGG TCG TA-3', mouse IL-1β sense, 5'-TGA AGG GCT GCT TCC AAA CCT TTG ACC-3'; anti-sense, 5'-TGT CCA TTG AGG TGG AGA GCT TTC AGG-3', human IL-6 sense, 5'-GCC TTC GGT CCA GTT GCC TT-3'; anti-sense, 5'-GCA GAA TGA GAT GAG TTG TC-3', mouse IL-6 sense, 5'-TGG AGT CAC AGA AGG AGT GGC TAA G-3'; anti-sense, 5'-TCT GAC CAC AGT GAG GAA TGT CCA C-3', human TNF-α sense, 5'-TGA GCA CTG AAA GCA TGA TC-3'; anti-sense, 5'-TTA TCT CTC AGC TCC ACG CC-3', mouse TNF-α sense, 5'-TTC TGT CCC TTT CAC TCA CTG G-3'; anti-sense, 5'-TTG GTG GTT TGC TAC GAC GTG G-3', human MCP-1 sense, 5'-CCC TTC TGT GCC TGC TGC TCA-3'; anti-sense, 5'-CTG TTC GTT TGG GTT TGA GGC TT-3', mouse MCP-1 sense, 5'-GAA GGA ATG GGT CCA GAC AT-3'; anti-sense, 5'-ACG GGT CAA CTT CAC ATT CA-3', human RANTES sense, 5'-CCT CGC TGT CAT CCT CAT TGC T-3'; anti-sense, 5'-TAC TCC CGA ACC CAT TTC TTC TC-3', mouse RANTES sense, 5'-GAT GGA CAT AGA GGA CAC AAC T-3'; anti-sense, 5'-TGG GAC GGC AGA TCT GAG GG-3', human IP-10 sense, 5'-ATC AAA CTG CGA TTC TGA TTT GCT GCC TTA-3'; anti-sense, 5'-TGG CCT TCG ATT CTG GAT AG-3', mouse IP-10 sense, 5'-ACC ATG AAC CCA AGT GCT GCC GTC-3'; anti-sense, 5'- GCT TCA CTC CAG TTA AGG AGC CCT-3', human GAPDH sense, 5'-GTG AAG GTC GGT AAC GG-3'; anti-sense, 5'-GAT GCA GGG ATG ATG TTC TG-3', mouse GAPDH sense, 5'-AAC TTT GGC ATT GTG GAA GG-3'; anti-sense, 5'-ACA CAT TGG GGG TAG GAA CA-3'.

### CD40 siRNA transfection

CD40 small interfering RNA (siRNA)-expressing vectors were generated using the Silencer Express Kit (Ambion Inc., Austin, TX). Sense (ACA CTA CAC AAA TGT TCC ACT GGG CTG AGA ACC GGT GTT TCG TCC TTT CCA CAA G) and anti-sense (CGG CGA AGC TTT TTC CAA AAA ATT CTC AGC CCA GTG GAA CAC TAC ACA AAT G) hairpin siRNA template oligonucleotides, specific for CD40 mRNA, were used [18].

Transfection was performed according to the manufacture's method. Briefly, 1 μg of vector expressing CD40 siRNA or control siRNA was incubated with 50 μl of serum-free media for 5 min (Solution A), and 2 μl Lipofectamine 2000 (Invitrogen, Carlsbad, CA) was incubated with serum-free media for 5 min (Solution B). Solution A was mixed with Solution B, and incubated for 20 min. After incubation, U87 cells were added to the mixture. The expression of CD40 after CD40 siRNA transfection was performed using western blot. Next, transfected-U87 cells were co-cultured with HMC-1 cells for various times. After co-culture, the [Ca^2+^]_i _levels, Rho families, PKC isoforms and MAP kinases were analyzed using a LSM 510 laser scanning microscopy, GST effector pull-down assay, Western blot, and EMSA, respectively.

### Glutathione-s-transferase (GST) effector pull-down assay

Small GTPase protein activities were assayed as previously described [18] using EZ-DetectTM protein Activation kits (Upstate Biotechnology, Lake Placid, NY). Co-cultured-astrocytes (1 × 10^7 ^cells) were suspended in 0.5 ml of a lysis buffer [25 mM Tris-HCl (pH 7.5), 150 mM NaCl, 5 mM MgCl_2_, 1% NP-40, 1 mM DTT, 5% glycerol, 1 mM PMSF, 1 μg/ml aprotinin, 1 μg/ml leupeptin (Roche Molecular Biochemicals, Mannheim Germany)] for 30 min on ice, and supernatants were obtained by centrifugation (13,000 × g for 20 min). The active form of small GTPase proteins were obtained according to the manufacturer's protocol from the supernatants by affinity precipitation using Pak-1 protein binding domain (PBD), which was fused to GST (glutathione-S-transferase), and visualized by Western blot analysis with anti-rabbit Rac1/2, cdc42 (1:1,000; PIERCE, Rockford, IL).

### Western blot analysis

Co-cultured-U87 cells, -primary astrocytes (3 × 10^6 ^cells/50 μl) or EAE brain tissues (50 mg) were homogenized in lysis buffer [10 mM HEPES (pH 7.9), 10 mM KCl, 0.1 mM EDTA, 0.1 mM EGTA, 1 mM DTT, 0.5 mM PMSF, 2.0 μg/ml aprotinin, 2.0 μg/ml leupeptin], and allowed to swell on ice for 30 min. Cell lysates (μg) were subjected to 8-10% sodium dodecyl sulfate (SDS)-polyacrylamide gel electrophoresis (PAGE) and transferred to nitrocellulose membranes (Amersham Biosciences, Buckinghamshire, UK). Membranes were washed with phosphate-buffered saline (PBS; Gibco, Carlsbad, CA) containing 0.1% Tween 20 (PBST), and then blocked for 1 h in PBST containing 5% skim milk (BD Bioscience, Sparks, MD). After washing the membranes with PBST, the membranes were treated with antibodies against actin, CD40, CD40L, PKC isoforms, ERK, JNK, p38, Jak1/2, STAT1, CBP and TNFR1 (Santa Cruz Biotechnology, Santa Cruz, CA), and then membranes were treated with p-PKC isoforms, p-ERK, p-JNK, p-p38, p-JAK1/2, p-ser727 STAT1, p-Tyr (Cell Signaling, Beverly, MA) diluted in PBST (1:1,000), and incubated for 60 min at room temperature. Membranes were washed with PBST, and treated with HRP-conjugated goat anti-mouse or HRP-conjugated rabbit anti-goat IgG (diluted to 1:5,000~1:10,000; Zymed Laboratory Inc. San Francisco, CA) in PBST for 60 min. After washing, the protein bands were visualized using electrogenerated chemiluminescent (ECL) solution (Amersham Biosciences, Buckinghamshire, UK) [18].

### Electrophoretic mobility shift assay (EMSA)

EMSA was performed with ^32^P-labed probes and 2 μg of nuclear extract in 40 μL of EMSA reaction buffer [18]. To perform the competition assay, a 100-fold excess of unlabeled competitor primer was added to the EMSA reaction mixture. Nuclear extracts were prepared from co-cultured cells (1 × 10^6 ^cells). Cells were washed twice with ice-cold PBS, and resuspended in 1 ml ice-cold buffer A [10 mM HEPES/KOH (pH 7.9), 10 mM KCl, 1.5 mM MgCl_2_, 0.5 mM DTT, 0.2 mM PMSF, 1 μg/ml leupeptin, and 1 μg/ml aprotinin]. After incubation on ice for 15 min, the cells were lysed by adding Nonidet P40 (10 μl 10% Nonidet P40, to a final concentration of 0.625%, v/v) and immediately vortexed for 10 sec. Nuclei were harvested by centrifugation at 20,000 × g for 1 min and resuspended in 40 μl ice-cold buffer C [20 mM HEPES/KOH (pH 7.9), 0.42 M NaCl, 1.5 mM MgCl_2_, 0.2 mM EDTA, 0.5 mM DTT, 25% glycerol, 0.2 mM PMSF, 1 μg/ml leupeptin, and 1 μg/ml aprotinin]. After incubation at 4°C for 20 min on a shaking platform, the nuclei were clarified by centrifugation at 15,000 × g for 10 min. The supernatant (nuclear extract) was then transferred to a new tube, and quantified using Bradford's method.

The 10 μl of a mixture of NF-κB (5'-AGT TGA GGG GAC TTT CCC AGG C-3', 3'-TCA ACT CCC CTG AAA GGG TCC G-5') or AP-1 (5'-CGC TTG ATG AGT CAG CCG GAA-3', 3'-GCG AAC TAC TCA GTC GGC CTT-5') oligonucleotide (1.75 pmol/μl; Promega Corporation, Madison, WI), T4 polynucleotide kinase 10 × buffer, [α-^32^P]ATP (10 μCi; 3,000 Ci/mmol; PerkinElmer, Waltham, MA), nuclear-free water, and T4 polynucleotide kinase (5~10 U/μl; Promega Corporation, Madison, WI) were incubated for 30 min at 37°C. The reaction was stopped by adding 1 μl EDTA (0.5 M). After adding 89 μl Tris-EDTA (TE) buffer [10 mM Tris-HCl (pH 8.0), and 1 mM EDTA], unincorporated nucleotides were removed from the DNA probe by chromatography through a G-25 spin column (Amersham Biosciences, Bucking-hamshire, UK). The nuclear extract and gel shift binding 5× buffer [20% glycerol, 5 mM MgCl_2_, 2.5 mM EDTA, 2.5 mM DTT, 250 mM NaCl, 50 mM Tris-HCl (pH 7.5), and 0.25 mg/ml poly (dI-dC) (Roche Molecular Biochemicals, Mannheim, Germany)] were incubated at room temperature for 10 min. Next, 20-30 fmol of ^32^P-labeled NF-κB oligonucleotide was added, and the solution was incubated at room temperature for 20 min. After incubation, 1 μl of 10 × gel loading buffer was added to each reaction. Reaction mixtures were electrophoresed on 6% polyacrylamide gels, and gels were analyzed using FLA-2000 (FUJIFILM, Tokyo, Japan).

### Immunoprecipitation (IP) for STAT1 on tyrosine 701 phosphorylation

IP before the determination of phosphorylation of STAT1 on tyrosine 701 using immunoblotting was performed according to method previously described [33]. Agarose conjugate (50 ml) was washed twice with washing buffer (PBS, pH 7.4), centrifuged for 10 sec at 12,000 × g at room temperature, and then resuspended in washing buffer. Agarose conjugate was added to 10 μl of anti-STAT1 antibody, incubated for 60 min at room temperature with gentle mixing, and then centrifuged at 3,000 × g for 2 min at 4°C. Samples were washed with 1 ml washing buffer, and centrifuged at 3,000 × g for 2 min at 4°C; this step was repeated at least twice. Co-cultured cell lysates (200 μg of protein) were added to agarose conjugate-bound antibody, and incubated overnight at 4°C with gentle mixing. Immunoprecipitated complexes were washed with washing buffer, and centrifuged at 3,000 × g for 2 min at 4°C. Pellets were washed with 1 ml washing buffer, and centrifuged at 3,000 × g for 2 min at 4°C. This step was repeated at least three times. The pellet was resuspended in 25-100 μl Laemmli sample buffer [0.125 M Tris HCl (pH 6.8), 4% SDS, 20% glycerol, 10% 2-mercaptoethanol, 0.004% bromophenol blue]. Samples were heated at 95°C for 5 min, centrifuged, and the supernatants were collected (IP sample). Samples were run on SDS-PAGE, transferred to nitrocellulose, and immunoblotting was performed.

### Induction of EAE

Female mice (C57BL/6, 8 weeks old) were purchased from Samtako BioKorea (Osan, Korea) and maintained in specific pathogen-free conditions before sacrifice. All mice were housed in accordance with guidelines from the Association for Assessment and Accreditation of Laboratory Animal Care (AAALAC), and all protocols were approved by the Institutional Review Board and conducted in the Laboratory Animal Research Center of Sungkyunkwan University.

The EAE model was induced by a method described previously [18]. Mice (8 mice/group) were divided into five groups: control, mice injected with CFA alone; EAE, mice received a subcutaneous injection of 150 μg myelin oligodendrocyte glycoprotein peptide 35-55 (MOG 35-55; Sigma, St. Louis, MO) in 100 μl PBS mixed with 100 μl of CFA (Sigma, St. Louis, MO); three treated groups, mice pretreated by intraperitoneal (i.p.) injection of anti-CD40 antibody (1 mg/kg), 8-oxo-dG (60 mg/kg), and a combination of both for 5 days after MOG injection, respectively. After MOG injection, each animal received an i.p. injection of 200 ng pertussis toxin (Invitrogen Life Technologies, Carlsbad, CA) in 200 μl PBS. The mice were weighed and scored daily in a blinded fashion by two examiners according to the following scale: score 0, no disease; score 1, loss of weight and tail weakness; score 2, weakness in hind limb; score 3, complete hind limb paralysis; score 4, hind limb paralysis with forelimb weakness or paralysis; and score 5, moribund or deceased. The concentration of anti-CD40 antibody (1 mg/kg) and 8-oxo-dG (60 mg/kg) was injected the same amount used in our previous experiments [18,34].

Thirty-two days after starting injection, the EAE score was about 3.8 ± 0.21, and brains were isolated, and inflammatory cells infiltrated into brain tissues were determined using hematoxilin and eosin (H&E) [18]. In general, EAE score reached peak on day 21- 25, but our EAE score reached peak on day 31- 32 despite the same method used in other laboratories [15,17]. This difference may be due to environmental factors. Brain tissues were fixed in 4% paraformaldehyde, embedded in paraffin, and cut into 3 μm sections. Brain sections were deparaffinized with xylene and washed using various percentages of ethanol. Endogenous peroxidase activity was blocked with 3% hydrogen peroxide in methanol for 5 min. Slides were then blocked with 1% BSA in PBS for 1 h. For immunohistochemistry, a polyclonal primary antibody against c-kit, GFAP, TNFR1 (Santa Cruz Biotechnology, Santa Cruz, CA; 1/50 dilution) was applied and the slides were incubated at 4°C for 24 h. After washing in PBS, slides were treated with biotinylated secondary antibody for 10 min, streptoavidin-HRP for 10 min, and chromogen substrate (DakoCytomation, Carpinteria, CA) for 5-10 min. For immunofluorescence, a polyclonal primary antibody against c-kit or GFAP was then applied and the slides were incubated at 4°C for overnight. After washing in PBS, slides were treated with the corresponding FITC or TEXAS-Red conjugated anti-IgG for 1 h at room temperature. After washing in PBS, the slides were mounted and examined using a confocal microscope (LSM 5 EXCITER, Carl Zeiss, Oberkochen, Germany) [18].

### Statistic analysis

**Figure 1:**
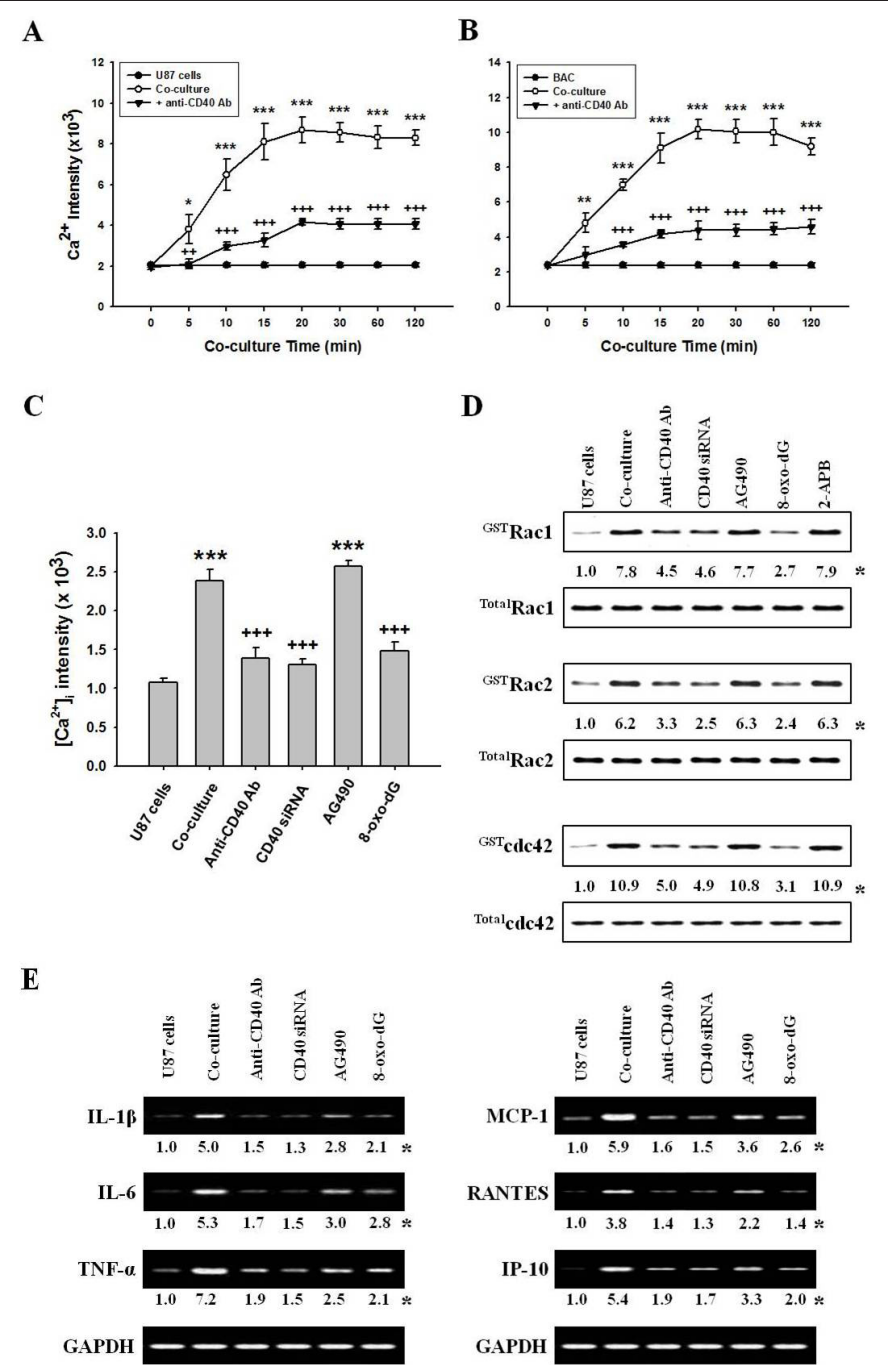
**Activation of astrocytes co-cultured with mast cells**. Astrocytes (3 × 10^6 ^cells) and mast cells (1 × 10^6 ^cells) were co-cultured in a 3:1 ratio. The anti-CD40 antibody (300 ng/ml), Jak inhibitor (10 μM AG490), 8-oxo-dG (300 μg/ml) or 2-APB (100 μM) was pretreated in astrocytes 1 h, 5, 10 and 10 min, respectively, before co-culture, and CD40 siRNA was transfected as described in "Methods". Fluro-3 AM (5.0 μM) was added to co-cultured-U87 cells or -primary astrocytes and incubated for 30 min. The [Ca^2+^]_i _level in U87 cells or in primary astrocytes was analyzed by confocal laser scanning microscopy. Rho-family GTPases or expressions of cytokine mRNA were determined in protein extracts and nuclear extracts by GST pull-down assay and RT-PCR, respectively. **(A, B) **Time course of [Ca^2+^]_i _level in co-cultured-U87 cells or -primary astrocytes, respectively, and in anti-CD40 antibody pretreatment. **(C) **The [Ca^2+^]_i _level after pretreatment with anti-CD40 antibody, CD40 siRNA or other inhibitors. **(D) **The activation of Rho-family GTPases by various inhibitors. (E) Expressions of cytokine mRNA caused by various inhibitors. U87 cells, U87 cell culture alone; BAC, primary astrocyte culture alone; Co-culture, U87 cells co-cultured with HMC-1 cells or BAC with BMMCs; Anti-CD40 Ab, anti-CD40 antibody pretreatment; CD40 siRNA, CD40 siRNA transfected-U87 cells co-cultured with HMC-1 cells; AG490, Jak inhibitor; 8-oxo-dG, 8-hydroxydeoxyguanosine. *, The numbers below bands are mean values and Figure 1A, B and C are mean ± SEM obtained from four independent experiments (n = 4) as the ratio bad density of each group versus that of total protein or GAPDH using densitometry analysis. *, *P *< 0.05; **, *P *< 0.01; ***, *P *< 0.001 versus U87 cell or primary astrocyte culture alone. ^++^, *P *< 0.01; ^+++^, *P *< 0.001 versus co-cultured-U87 cells or -primary astrocytes.

Experimental data are shown as means ± standard error of mean (SEM). The unpaired Student's *t*-test was used to analyze the results for statistical significance when only two conditions were compared. P values below 0.05, 0.01, or 0.001 were considered significant. The densitometry analysis of immunoblots, PCR and EMSA was performed with Quantity One version 4.6.3 (BIO-RAD, Hercules, CA), numbers below bands in all figures are mean obtained from four independent experiments (n = 4) as the ratio of band density of each group versus that of total protein or loading control, and the variable percentile among four independent experiments was less than 10%. Histogram for densitometry analysis in the [Ca^2+^]i level in Figures 1A, B and C or Figure 5B was indicated by mean ± SEM (n = 4) obtained from four independent experiments.

## Results

### Intracellular Ca^2+ ^([Ca^2+^]_i_) levels in co-cultured-astrocytes

Astrocytes secrete many kinds of bioactive substances including growth factors and cytokines. These secretions are mediated by Ca^2+^-dependent system, which may play important roles in the regulation of neuronal and brain functions [35]. Therefore, we observed the [Ca^2+^]_i _level in the co-culture of U87 cells and HMC-1 cells or co-culture of primary astrocytes and bone marrow-derived mast cells (BMMCs) (hereafter refer to co-cultured-U87 cells or co-cultured-primary astrocytes; Additional file 1 Figure S1A). The [Ca^2+^]_i _levels increased in a time-dependent manner in both the co-cultured-U87 cells (Figure 1A) and co-cultured-primary astrocytes (Figure 1B). The [Ca^2+^]_i _levels maximized at 20 min in both the co-cultured-U87 cells and co-cultured-primary astrocytes.

### Effects of anti-CD40 antibody or CD40 siRNA on [Ca^2+^]_i _levels in co-cultured-astrocytes

Our previous study suggested that astrocytes and mast cells may cross-talk through CD40-CD40L interaction, as supported by the report that co-cultured-astrocytes (U87 cells) enhanced expression of CD40 molecules [18]. However, CD40L was not detected in co-cultured-U87 cells, co-cultured HMC-1 cells showed higher levels of CD40L and similar levels of CD40 molecules compared to the control (Additional file 1 Figure S1B).

Therefore, we observed that whether anti-CD40 antibody decreased [Ca^2+^]_i _levels in the co-cultured-U87 cells (Figure 1A) and co-cultured-primary astrocytes (Figure 1B) in a time-dependent manner, but did not completely inhibit [Ca^2+^]_i _levels in either co-cultured-astrocytes. CD40 siRNA, which confirmed the expression of CD40 after CD40 siRNA transfection (Additional file 2 Figure S2A), or 8-oxo-dG (8-hydroxydeoxyguanosine), which is a Rac1/2 and cdc42 inhibitor [34], also decreased [Ca^2+^]_i _levels in co-cultured-U87 cells (Figure 1C).

### Effects of anti-CD40 antibody, CD40 siRNA or 8-oxo-dG on cytokine expressions in co-cultured-U87 cells

We previously reported that cytokine protein and mRNA expression [IL-1β, IL-4, IL-5, IL-6, IL-8, IL-9, IL-10, TNF-α, monocyte chemotactic protein-1 (MCP-1), regulated on activation normal T cell expressed and secreted (RANTES), interferon-gamma-induced protein-10 (IP-10), and monokine induced by IFN-gamma (MIG)] were secreted into the co-cultured media and expressed in co-cultured-mast cells, respectively [18]. The cytokine mRNAs such as ones for IL-1β, IL-6, TNF-α, MCP-1, RANTES, and IP-10 were also increased in both co-cultured-U87 cells and -primary astrocytes (Additional file 1 Figure S1C). Anti-CD40 antibody, CD40 siRNA or 8-oxo-dG pretreatment prevented this increase in cytokine mRNA levels in the co-cultured-U87 cells (Figure 1E).

### Effect of anti-CD40 antibody, CD40 siRNA or 8-oxo-dG on the various signaling molecules in co-cultured-U87 cells

Rho-family GTPases modulate Ca^2+^-dependent ATP release from astrocytes [36]. Similarly, we observed that Rho-family GTPase (Rac1, Rac2 and cdc42) activities reached a maximum at 20 min in co-cultured-U87 cells or -primary astrocytes (Additional file 2 Figure S2B). Anti-CD40 antibody, CD40 siRNA or 8-oxo-dG blocked the increase of these Rho family activities in co-cultured-U87 cells (Figure 1D).

**Figure 2:**
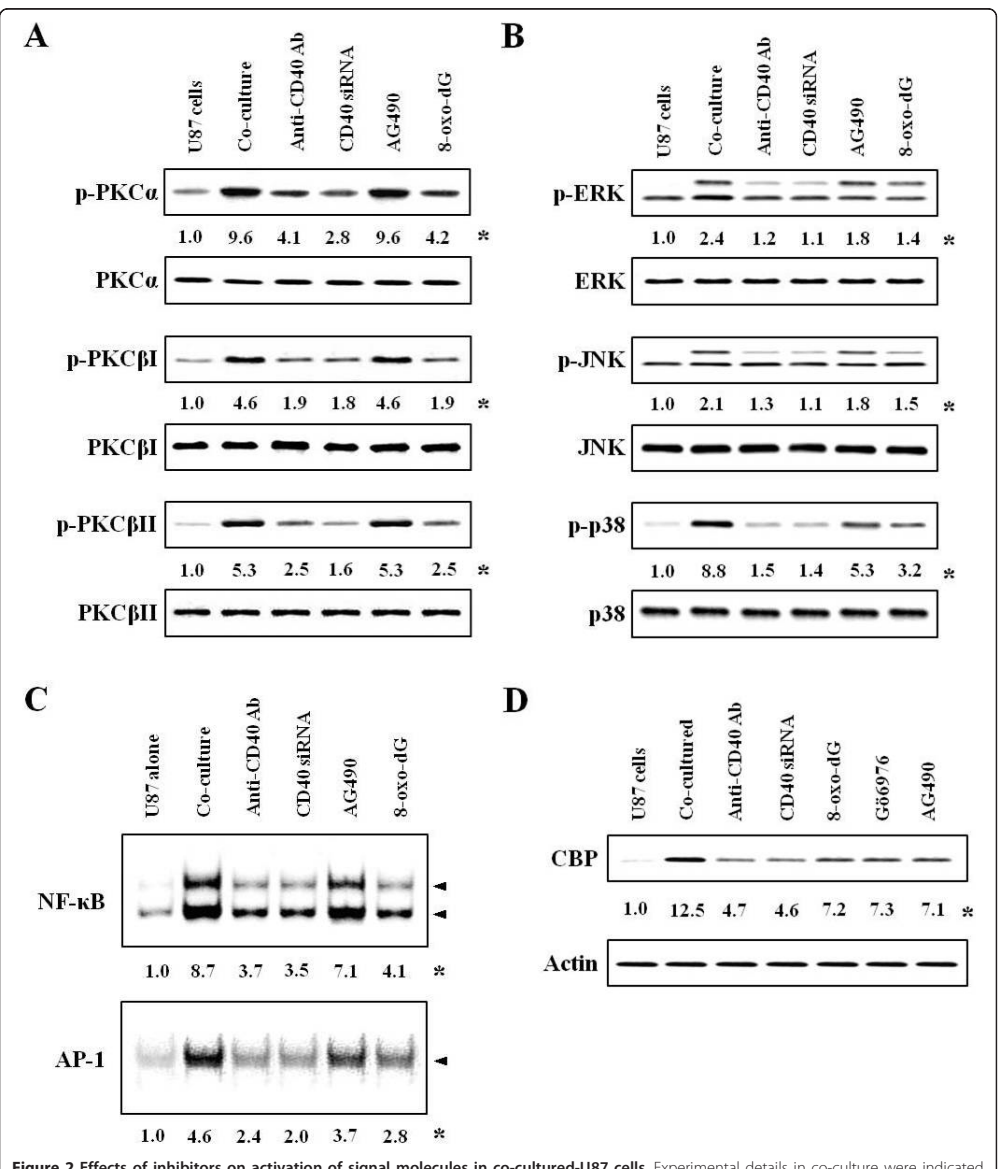
**Effects of inhibitors on activation of signal molecules in co-cultured-U87 cells**. Experimental details in co-culture were indicated in Figure 1. The anti-CD40 antibody (300 ng/ml), Jak inhibitor (10 μM AG490) or 8-oxo-dG (300 μg/ml) was pretreated in astrocytes 1 h, 5 and 10 min, respectively, before co-culture, and CD40 siRNA was transfected, as described in "Methods". Activities of PKCs, MAP kinases and transcription factors were determined in protein extracts and nuclear extracts by Western blot and EMSA, respectively. **(A, B) **Activities of PKC isoforms and MAP kinases after inhibitor pretreatment. **(C, D) **Activities of NF-κB (upper panel) or AP-1 (lower panel) and expression of CBP after inhibitor pretreatment. *, Numbers below bands are mean values obtained from four independent experiments (n = 4) as the ratio of band density of each group versus that of total protein or actin using densitometry analysis.

**Figure 3:**
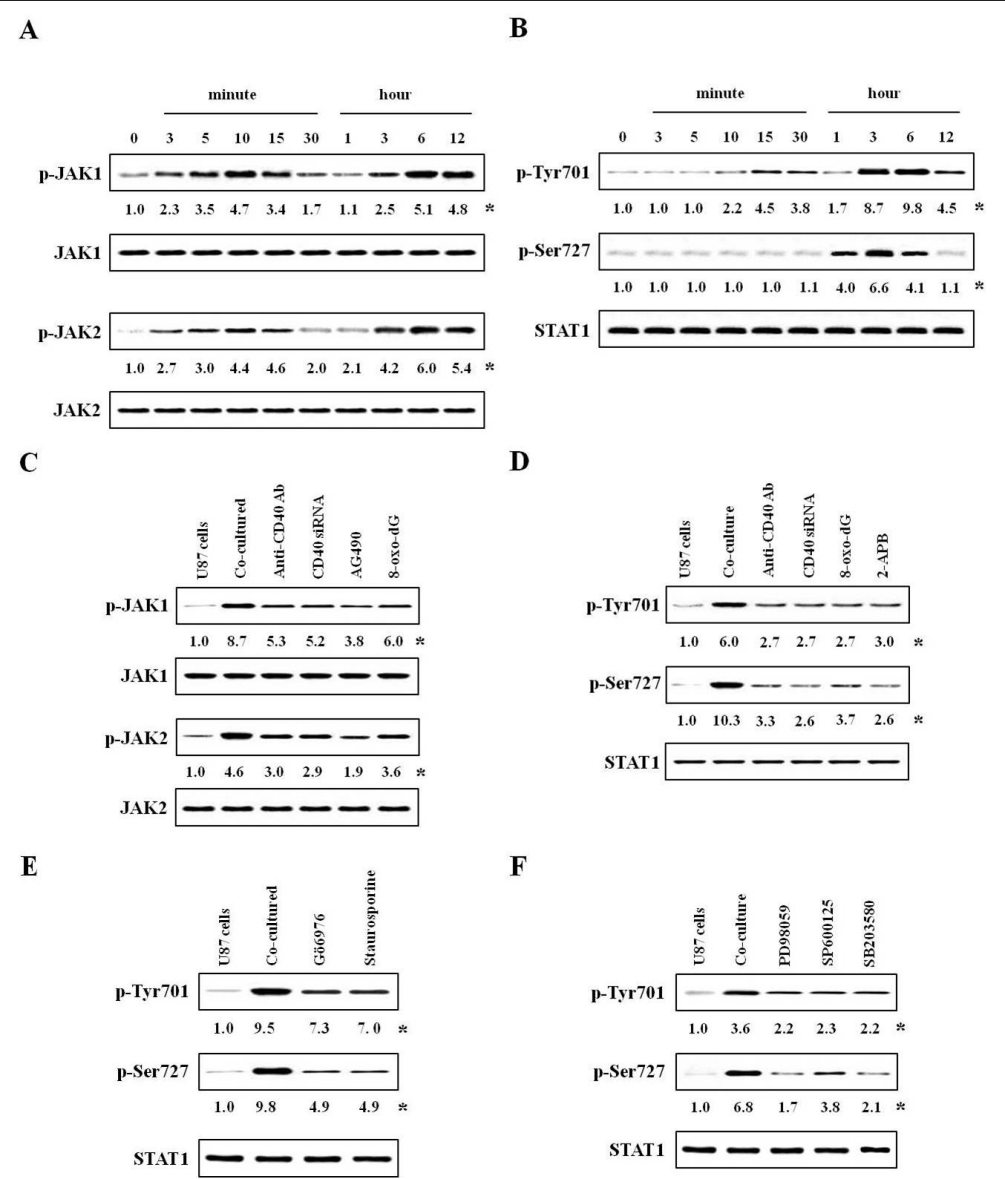
**Effects of inhibitors on activation of Jak 1/2 or STAT1 in co-cultured-U87 cells**. Experimental details in co-culture were indicated in Figure 1. The anti-CD40 antibody (300 ng/ml), Jak inhibitor (10 μM AG490), 8-oxo-dG (300 μg/ml), PKC inhibitors (5 nM Gö6976 or staurosporine) or inhibitors of MAP kinase (50 μM PD98059 for ERK, 10 μM SP600125 for JNK, 10 μM SB203580 for p38) was pretreated in astrocytes 1 h, 5, 10 or 10 min, respectively, before co-culture, and CD40 siRNA was transfected, as described in "Methods". Activities of JAKs and STAT1 were determined in protein extracts by western blot using densitometry analysis. **(A, B) **Time courses for activities of Jak 1/2 and STAT1 after co-culture. **(C, D) **Activities of Jak 1/2 and STAT1 after inhibitors pretreatment. **(E, F) **Phosphorylation of STAT1 after pretreatment with PKC and MAP kinase inhibitors. *, Numbers below bands are values obtained from four independent experiments (n = 4) as the ratio of band density of each group versus that of total protein using densitometry analysis.

Rac1 increases Ca^2+ ^influx in epithelial cells [37]. We confirmed cascades of signal pathways in co-cultured-astrocytes by observing that 8-oxo-dG inhibited [Ca^2+^]_i _levels (Figure 1C) as well as Rac1/2, cdc42 activation, but Ca^2+ ^inhibitor (2-aminoethoxydiphenyl borate, 2-APB) did not inhibit Rho family activities (Figure 1D). We also observed that activities of downstream molecules such as PKC isoforms, MAP kinases and transcription factors reached a maximum at 30 min, 1 h and 3 h, respectively, in the co-cultured-U87 cells and -primary astrocytes (Additional file 2 Figure S2C, D and Figure 3). However, the activities of other PKC isoforms (δ, ζ and λ) were not affected in either co-cultured-astrocytes (data not shown). 8-oxo-dG as well as anti-CD40 antibody and CD40 siRNA inhibited phosphorylation of PKC isoforms and MAP kinases (Figure 2A, B), and activities of transcription factors NF-κB and AP-1 (Figure 2C). Jak inhibitor (AG490) did not inhibit PKC isoforms (Figure 2A) and weakly inhibited the phosphorylation of MAP kinases (Figure 2B). The order of signal cascades was Rho-family GTPases, [Ca^2+^]_i_, PKCs and MAP kinases in accordance with time sequence as reported previously in co-cultured-mast cells [18].

Since CREB-binding protein (CBP) functions as a co-activator for various transcription factors including signal transducers and activators of transcription STAT1 on serine 727 (STAT1^727^) and NF-κB [38], we examined whether CBP showed STAT1- and NF-κB-dependent transcriptional synergy. CBP expression was increased in co-cultured-U87 cells and decreased by various inhibitors (Figure 2D). This data demonstrated that CBP was mediated by Rho-family GTPase/PKCs/NF-κB and STAT^727 ^pathways.

### Phosphorylations of Janus kinase 1 (Jak1) and Jak2 or expression of STAT1 in co-cultured-U87 cells

Jak/STAT signal pathways play a critical role in the cytokine-dependent stimulation of astroglial cells [39], and as presented in Figure 1E, co-cultured-astrocytes expressed cytokines mRNAs. Therefore, we examined their signal pathways for cytokines expression. Interestingly, phosphorylation of both Jak1/2 and STAT1 on tyrosine 701 (STAT1^701^) showed diphasic increase in co-cultured-astrocytes. That is, the phosphorylation of Jak1/2 and STAT1^701 ^were initiated at 3 min and 10 min, and reached at a maximum 10 min and 15 min, respectively. And, their phosphorylation was strongly induced and maximized at 6 h after co-culture (Figure 3A, B). However, the phosphorylation of STAT1^727 ^only reached a maximum at 3 h in co-cultured-U87 cells (Figure 3B).

### The effect of inhibitors on Jak1/2 and STAT1 in co-cultured-U87 cells

To confirm the signal cascade downstream of Jak/STAT1, we used various inhibitors. First, we observed that phosphorylation of Jak1/2 was inhibited by anti-CD40 antibody, CD40 siRNA or Rac inhibitor 8-oxo-dG as well as Jak1/2 inhibitor AG490 (10 μM) (Figure 3C). The Jak inhibitor (AG490) was not effective on [Ca^2+^]_i _level (Figure 1C) and small GTPases (Figure 1D).

Anti-CD40 antibody, CD40 siRNA or 8-oxo-dG inhibited phosphorylation of both STAT1^701 ^and STAT1^727^. The Ca^2+ ^influx inhibitor (2-APB) inhibited STAT1^701 ^and STAT1^727 ^(Figure 3D). However, with pretreatment of these inhibitors, STAT1^727 ^activity downstream of Rho-family and Ca^2+ ^signals was reduced by a much greater degree compared to STAT1^701 ^activity. This phenomenon inferred that STAT^701 ^is not downstream of Ca^2+ ^signals, but it is indirectly evoked by inhibiting the Ca^2+ ^pathway via Rho-family

A PKCα- and βI-specific inhibitor (5 nM Gö6976) and non-specific inhibitor (5 nM staurosporine), or all inhibitors of MAP kinases (50 μM PD98059, 10 μM SP600125, 10 μM SB203580) remarkably inhibited the phosphorylation of STAT1^727^, but weakly inhibited STAT1^701 ^activity (Figure 3E, F).

To elucidate the signaling cascades of PKC and MAP kinase, we used inhibitors of PKCs and MAP kinases, although the order of their cascades was observed over the time courses for the above activities (Additional file 2 Figure S2C, D and Figure 3A). These results showed that MAP kinases are downstream of PKC isoforms (Additional file 3 Figure S3B, C) as reported previously in co-cultured-mast cells [18]. Additionally, PKC inhibitors and MAP kinase inhibitors reduced the activities of transcriptional factors or cytokine expression (Additional file 4 Figure S4A, B).

### Effects of TNF receptor 1 (TNFR1) antibody on activation of co-cultured-U87 cells

**Figure 4:**
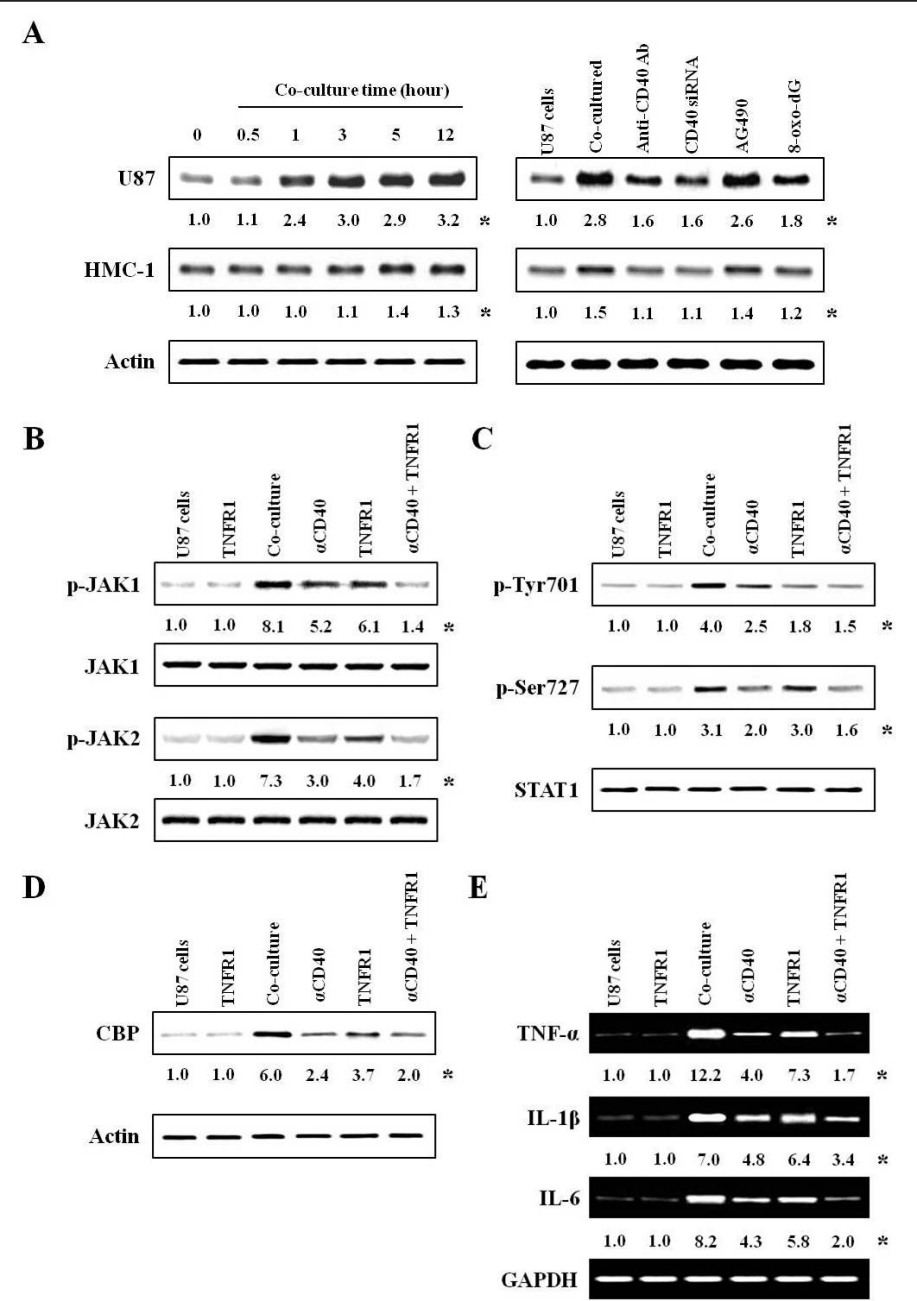
**The effects of anti-TNFR1 antibody on the activation of co-cultured-U87 cells**. Experimental details in co-culture were indicated in Figure 1. The anti-TNFR1 antibody (300 ng/ml) or anti-CD40 antibody (300 ng/ml) was pretreated in astrocytes 30 min and 1 h before co-culture. Expressions of TNFR1, activities of JAKs, STAT1 and CBP, and expressions of cytokine mRNA were determined in protein extracts and nuclear extracts by western blot and RT-PCR, respectively. **(A) **Expression of TNFR1 in co-cultured-U87 (left-upper panel) or -HMC-1 cells (left-lower panel), and expression of TNFR1 after inhibitor pretreatment (right panel). **(B, C) **Phosphorylations of Jak1/2 and STAT1 after inhibitor pretreatment. **(D) **Expression of CBP after inhibitor pretreatment. **(E) **Expressions of cytokine mRNA after inhibitor pretreatment. U87 cells, U87 cell culture alone; TNFR1, anti-TNFR1 antibody alone pretreatment in U87 cells; Co-culture, U87 cells co-cultured with HMC-1 cells; αCD40, anti-CD40 antibody pretreatment; αCD40 + TNFR1, anti-CD40 antibody and anti-TNFR1 antibody pretreatment before co-culture. *, Numbers below bands are values obtained from four independent experiments (n = 4) as the ratio of band density of each group versus those of total protein, actin or GAPDH using densitometry analysis.

Since various cytokines were secreted in the co-culture system and Jak/STAT1^701 ^were activated by diphasic events, we inferred that cytokines secreted from co-cultured-astrocytes may re-activate astrocytes. Thus, we targeted TNF-α which is secreted by both co-cultured-astrocytes and -mast cells and is also related to neurodegeneration and chronic inflammation in astrocytes [40]. First, we observed that TNF-α receptor 1 (TNFR1) expression reached a maximum at 3 h in the co-cultured-U87 cells (Figure 4A; left-upper panel). However, this was only weakly enhanced in co-cultured-HMC-1 cells and reached a maximum at 5 h (Figure 4A; left-lower panel). Receptor expression was strongly inhibited by anti-CD40 antibody, CD40 siRNA or 8-oxo-dG in co-cultured-U87 cells, but Jak inhibitor (AG490) did not reduce (Figure 4A; right panel) expression.

Anti-TNFR1 antibody (300 ng/ml) pretreatment suppressed activities of Jak1/2 (Figure 4B) and STAT1 (Figure 4C), and CBP expression (Figure 4D). TNFR1 antibody inhibited activity of STAT1^701 ^downstream of Jak signal cascades more than that of STAT1^727 ^(Figure 4C). Anti-TNFR1 antibody also suppressed expression of IL-1β and IL-6 mRNA as well as TNF-α mRNA expressed in co-cultured-U87 cells (Figure 4E). Optimal time (30 min) and concentration (300 ng/ml) for inhibition by anti-TNFR1 antibody were determined in the preliminary experiments (Additional file 5 Figure S5A, B).

### Clinical EAE score and co-localization of TNFR1 and astrocyte surface marker in EAE-induced brain tissues

**Figure 5:**
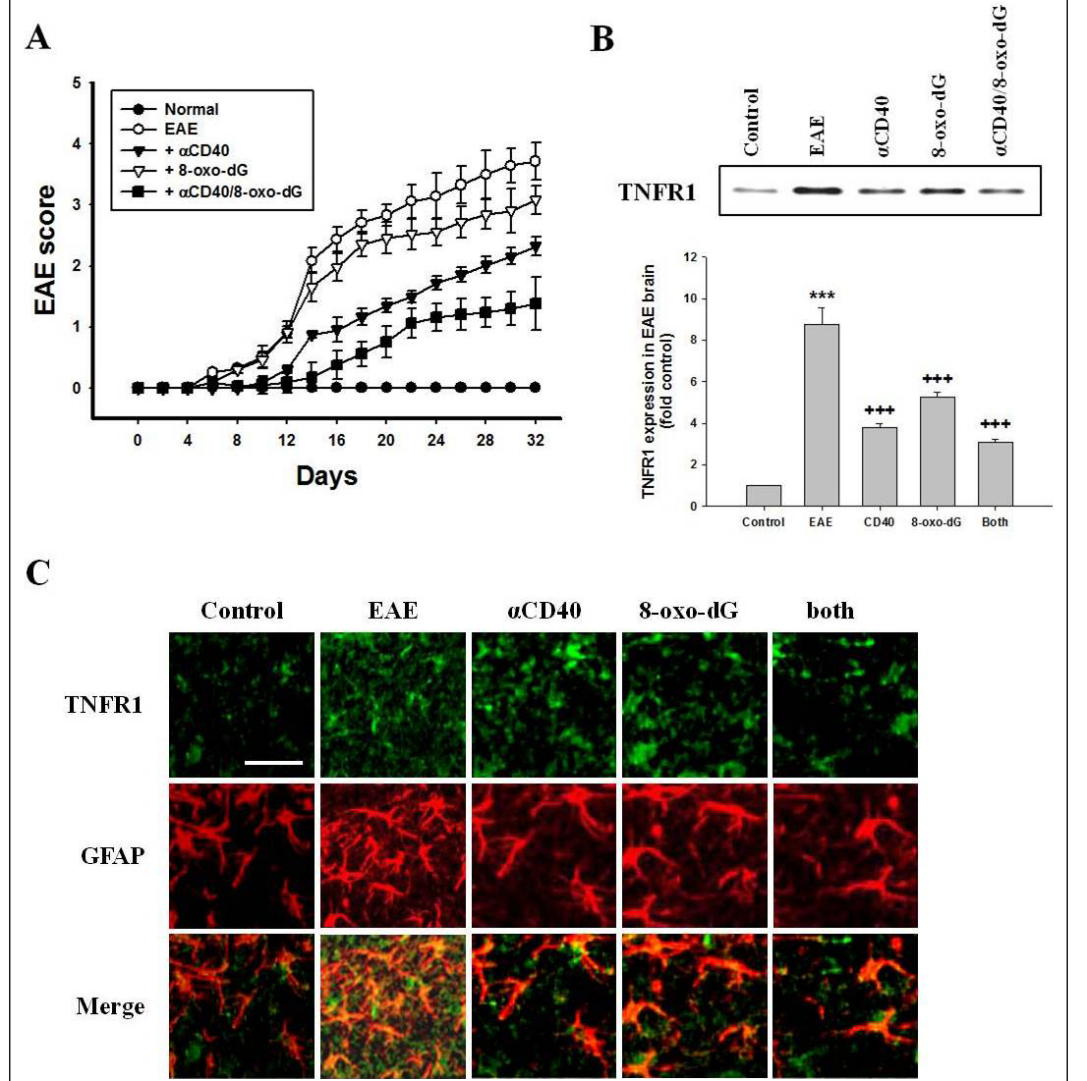
**Clinical score and co-localization of TNFR1 in EAE-induced mouse brain**. EAE mouse model was immunized with MOG and CFA, and scored daily as described in "Methods". Mice received anti-CD40 antibody (1 mg/kg), 8-oxo-dG (60 mg/kg) or a combination of anti-CD 40 antibody and 8-oxo-dG, respectively, with i.p. injection for 5 days after MOG injection. After animal sacrifice at day 32 (EAE score, 3.8 ± 0.21), brains were removed and preserved in 10% neutral buffered formalin. Expressions of TNFR1 were determined in protein extracts by western blot, and co-localization was determined by double staining for TNFR1 (green) and GFAP (red). **(A) **EAE score after pretreatment with inhibitors Data are mean ± SEM (n = 8). **(B) **Expression of TNFR1 (n = 4). Histogram for densitometry analysis was indicated by mean ± SEM (n = 4) obtained from four independent experiments. **(C) **Co-localization of TNFR1 and astrocytes after pretreatment with inhibitors (n = 4). The image depicted one of representative results after four independent experiments were determined. TNFR1, TNF-α receptor 1; αCD40, anti-CD40 antibody; 8-oxo-dG, 8-hydroxydexoyquanosine; both, a combination of anti-CD40 antibody and 8-oxo-dG. Bar in control group indicates 100 μm.

In our data, EAE score (3.8 ± 0.21) maximized on days 32, and inflammatory cells were remarkably infiltrated into brain tissues (Additional file 6 Figure S6A). Anti-CD40 antibody significantly reduced EAE score, but 8-oxodG weakly inhibited. Both treatments reduced more than additive effect of each inhibitor (Figure 5A).

It has been suggested that TNF-α plays a pivotal role in the pathogenesis of inflammatory demyelinating disease in MS [26,41] and EAE models [18,42]. Therefore, we investigated the expression of TNFR1 in the EAE model (Figure 5B). In the EAE thalamus co-localized with mast cells and astrocytes, TNFR1 level was remarkably enhanced. This enhancement of cytokine receptor was observed more frequently in astrocytes than in mast cells. Pre-treatment with anti-CD40 antibody, 8-oxo-dG, or a combination of both compounds decreased TNFR1 expression (Figure 5B).

Next, we investigated co-localization of TNFR1 and surface molecule of astrocytes or mast cells in the brain of the EAE model. TNFR1 expression (green) and GFAP (red) was enhanced in astrocytes in EAE brain tissues. Co-localization of TNFR1 and GFAP (yellow) was enhanced in astrocytes double-labeled with GFAP (red) and TNFR1 (green) in the EAE (Figure 5C). In double-labeling with c-kit (red) and TNFR1 (green) in brain tissues, TNFR1 expression was enhanced in EAE brain tissues, but co-localization of TNFR1 and c-kit was enhanced weaker than surface markers of astrocytes (Additional file 6 Figure S6). Anti-CD40 antibody or 8-oxo-dG reduced expression of TNFR1 in astrocytes in the brain of the EAE model, and a combination of both compounds inhibited TNFR1 expression more than use of each agent alone.

**Figure 6:**
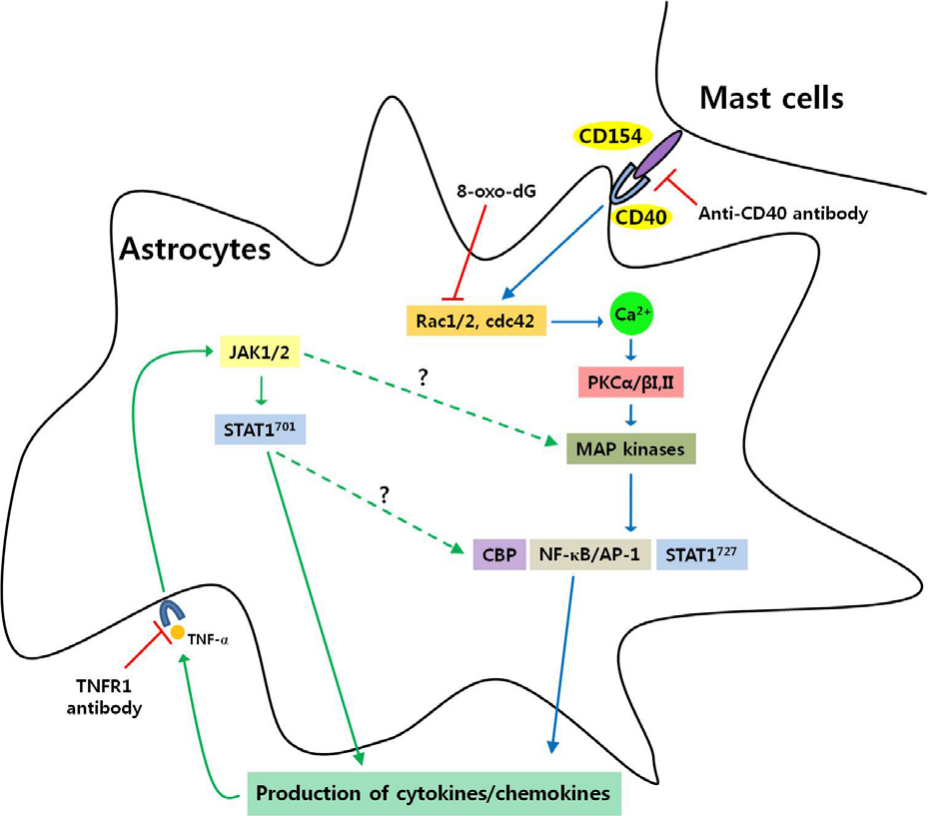
**Schematic diagram showing signaling pathways in co-cultured-astrocytes**. The data suggest that activation of astrocytes caused by co-culturing with mast cells through CD40-CD40 interaction mainly induces production of cytokines and chemokines via Rho-family GTPases/Ca^2+^-dependent PKC isoforms, MAP kinases, NF-κB and STAT1^727^. These cytokines subsequently re-activate astrocytes, and enhance the production of a variety of cytokines via Jak/STAT1^701 ^or STAT1^727^/CBP pathways. TNFR1, TNF-α receptor 1; CD154, CD40 ligand. Broken lines show parts of the pathways requiring further study.

We produced schematic diagrams showing signaling pathways in the activation of astrocytes through CD40-CD40L interaction in co-culture with mast cells (Figure 6). This diagram suggest that activation of astrocytes caused by co-culturing with mast cells through CD40-CD40 interaction mainly induces production of cytokines and chemokines via Rho-family GTPases/Ca^2+^-dependent PKC isoforms, MAP kinases, NF-κB and STAT1^727^. These cytokines subsequently re-activate astrocytes, and enhance the production of a variety of cytokines via Jak/STAT1^701 ^or STAT1^727^/CBP pathways.

## Discussion

This study demonstrated that astrocytes are activated by interaction of CD40-CD40L in a co-culture system with mast cells. The activated astrocytes induced production of cytokines (IL1β, IL-6, TNF-α, mCP-1, RANTES, IP-10) through Rho-family/Ca^2+^/PKC isoforms/MAP kinases/NF-κB-STAT1^727 ^signal pathways, which in turn re-activate astrocytes via the Jak/STAT1^701 ^signal pathways. Anti-CD40 antibody or CD40 siRNA inhibited all signal cascades via small GTPases, and anti-CD40 antibody or 8-oxodG reduced the EAE score and TNFR1 expression in EAE brain. Therefore, our data suggest that astrocytes activated by cell-to-cell contact, particularly with mast cells, may exacerbate the development of neurodegenerative disease including demyelization, such as MS, due to enhancement of cytokine receptor expression on astrocytes caused by inflammatory cytokine secretion as well as interaction of CD40 with CD40L in vitro and in mouse EAE model.

Mast cells accumulate in MS plaques [6,7] and in EAE brain [18,19,21]. Mast cells are activated by CD40-CD40L interaction in a co-culture with astrocytes, and both cells surface markers are enhanced and co-localized in EAE brain tissues [18], although it has been reported that mast cells are dispensable for the development of EAE [20]. Thus, the interaction between CD40 and CD40L plays an important role in signal transduction pathways in humoral and cell-mediated immune responses. CD40-CD40L interaction produces high levels of proinflammatory cytokines in immune cells of the CNS, including microglia and astrocytes [43,44]. During brain inflammation, astrocytes also are producers of a variety of cytokines including IL-1, IL-6, TNF-α, IL-10 and TGF-β, and chemokines attracting T cells within the CNS [23,44]. A variety of exocytotic mediators released from astrocytes influences neuronal development, function and plasticity [45]. Our data showed that these released cytokines are produced in astrocytes activated through CD40-CD40L interaction in the co-culture system (Figure 1E), as demonstrated by other laboratories that the appearance of CD40 in the CNS correlates with the expressions of inflammatory cytokines [23,44]. However, secretory pathways and the involved molecular mechanisms in astrocytes are poorly understood.

Activation of astrocytes, which provides support for neuronal function in the healthy and inflamed CNS [2], is usually manifested as a rise of intracellular Ca^2+ ^([Ca^2+^]_i_) level due to release of Ca^2+ ^from internal stores as well as Ca^2+ ^uptake from the extracellular space [45,46]. Thus, in order to clarify signal pathways for the production of cytokines induced in co-cultured-astrocytes, we first confirmed that a rise of [Ca^2+^]_i _level is induced through interaction of CD40 with CD40L in adjacent cells (Figure 1A, B).

Rho-family GTPases activate intracellular kinase cascades to modulate gene transcription, and participate in regulated secretory pathways [47], while Rac1 contributes to activation of STAT1 in astrocytes [48]. Our data suggest that Rho-family GTPases up-regulated downstream [Ca^2+^]_i _levels in co-cultured-astrocytes as Rac inhibitor (8-oxo-dG) reduced [Ca^2+^]_i _levels (Figure 1C), but the [Ca^2+^]_i _inhibitor (2-APB) did not inhibit Rac-family activity in co-cultured-astrocytes (Figure 1D).

Ca^2+^-dependent PKC and MAP kinase are the main signaling pathways involved in the synthesis and secretion of mediators [49]. MAP kinase components, such as ERK1/2, have an important role in astrocyte activation [23,50]. Astroglial reactivity, which is associated with the production of NF-κB-dependent proinflammatory molecules, is also an important component of the pathophysiology of chronic neurological disorders [22,24]. Additionally, phosphorylation of STAT1 on serine 727 (STAT^727^) independent of tyrosine phosphorylation (STAT^701^), which is activated downstream of PKCs and MAP kinases, is required to enhance transcriptional activity in various cells [48]. Therefore, our data inferred that astrocytes can be directly activated by CD40-CD40L interaction in co-culture, and that CD40-CD40L interaction mainly mediates signal cascades via Rho-family GTPases (Rac1/2, cdc42), [Ca^2+^]_i _levels, PKCs (α, βI, βII), MAP kinases, transcription factors (NF-κB or AP-1) and STAT1^727 ^(Figure 1A, B, C, D). This is supported by our data showing that phosphorylation of STAT1^727 ^functioned as a downstream regulator of PKCs (Figure 3C) and MAP kinases, and that the phosphorylation of STAT1^727 ^was inhibited by Rac, Ca^2+^, PKCs, MAP kinase inhibitors; however, Rho-family GTPases, [Ca^2+^]_i_, and PKCs were not inhibited by Jak inhibitor (Figures 1C, D).

Pretreatment with anti-CD40 antibody or CD40 siRNA significantly attenuated cytokine production and activation of signal molecules in the co-culture system, but did not completely inhibit. This implies that inflammatory cytokines secreted by cell-to-cell interaction of both cell surfaces may re-activate each other or that other signal pathways maybe exist. There are also reports that Jak/STAT^701 ^signaling pathway is involved in early events of cytokine stimulation in astrocytes [39], and that various cytokines and their receptors are expressed via the Jak/STAT1^701 ^pathway in brain section of patients with MS [51]. Therefore, we focused on the Jak/STAT^701 ^cytokine signaling pathway. Jak/STAT1^701 ^was not involved in Rac/Ca^2+^/PKCs pathways (Figures 1C, D and 2A). Activities of Jak/STAT^701 ^showed diphasic responses (Figure 3A, B). It can be inferred that Jak/STAT1^701^, which is weakly activated early (peak activity at 3 and 10 min, respectively) after co-culturing, is induced by interaction of CD40-CD40L. And, our data also infer that Jak/STAT^701^, which is strongly activated late (peak activity at 6 h) after co-culturing, is evoked by cytokines secreted via the Rho-family pathway. Therefore, our data suggest that cytokines produced in co-cultured-astrocytes are mainly induced by signaling via Ca^2+^/PKCs/MAP kinases/STAT1^727 ^downstream of Rho-family GTPases, and cytokine-induced astrocyte re-activation leads to further cytokine production via the Jak/STAT1^701 ^pathway. Evidence of this event is supported by our data that anti-TNFR1 antibody as well as anti-CD40 antibody suppressed activation of Jak/STAT1^701 ^and induction of cytokine mRNAs in co-cultured-astrocytes. This indicates that TNF-α bound to TNFR1 re-activates astrocytes via the Jak/STAT^701 ^pathway (Figure 4). Also, the reason why we chose TNF-α among the various cytokines secreted by co-cultured-astrocytes is that the TNF-α produced by astrocytes plays multiple roles in the development of neurological disorders [40] including MS [26] and EAE models [52,53] and the induction of other inflammatory cytokines, such as IL-1β and IL-6 etc. and chemokines [42]. Furthermore, overexpression of IL-1β and IL-6 in the CNS is also correlated with chronic active plaques in MS [54] and the development of EAE [27]. In showing that expression of IL-1β and IL-6 mRNA was inhibited by TNFR1 antibody (Figure 4E), our data are consistent with reports from other laboratories [42,55]. MCP-1 and IP-10 expressed in co-cultured-astrocytes also recruit leukocytes and provoke more inflammation [56].

STAT1 and NF-κB, which are integral transcription factors functioning in the regulation of genes involved in immune and inflammatory reactions, were shown to bind to the N-terminal and the C-terminal regions of CBP [57,58]. In the present study, the increased CBP expression was inhibited by various inhibitors of CD40, Rac, PKC, Jak and TNFR1 (Figure 2D). These data suggest that CBP is activated by two pathways (Jak/STAT^701 ^and Rho-family GTPasea/NF-κB-STAT1^727^).

We previously reported that mast cell population and co-localization of astrocytes and mast cells were increased in the thalamus of the EAE model [18]. Now, we demonstrated that TNFR1 expression was enhanced in co-cultured-astrocytes and thalamus of EAE-induced brain tissues. Co-localization of TNFR1 and astrocyte surface marker was also enhanced in the EAE-induced brain, and their co-localization and EAE score were reduced by anti-CD40 antibody or 8-oxo-dG administration (Figure 5). MS is a chronic and demyelinating disease affecting the white matter of the CNS, and an accumulation of mast cells in MS plaque was mainly increased in the demyelinated area i.e. the white matter [10]. However, the reason why we observed TNFR1 expression in thalamus is that mast cells are abundant in the thalamus, and considerable numbers of them are in the hypothalamus and median eminence in rat EAE model [59] and enhanced in thalamus and meninges of GFAP-IL3 mice in CNS demyelination, and that this study focused on the interaction of astrocytes and mast cells [60]. Therefore, we can infer that alteration of TNFR1 expression may be related to clinical manifestation of EAE, thus anti-CD40 antibody may attenuate the development of EAE in mice. That is, the data suggest that astrocytes and mast cells may directly interact in close proximity in the thalamus and produce inflammatory cytokines, and that EAE-related-cytokines secreted by cell-to-cell interaction re-activate each other, particularly astrocytes, and then enhance the expression of cytokine receptor and release more mediators including cytokines that may contribute to exacerbating the development of demyelination in neurodegenerative disease like MS. Therefore, it seems to us that a combination of anti-CD40 antibody and TNFR1 blockers may need for neurodegenerative disease therapy like MS. However, further study is needed to fully understand the role of CD40-CD40L interaction in the EAE model and their potential as therapeutic targets.

## Conclusions

The present study demonstrated that astrocytes activated through CD40-CD40L interaction in a mast cell co-culture system produce pro-inflammatory cytokines through Rho-family GTPases/Ca^2+ ^mobilization/PKCs/MAP kinases and NF-κB or STAT1^727 ^pathways, and the produced cytokines subsequently re-activate astrocytes via Jak/STAT1^701^. This study suggests that cell-to-cell contact between both types of cells is bi-directionally activated in vitro and in EAE model, and that both types of activated cells may initiate development of neurodegenerative diseases through various mediators. Furthermore, our data suggest that the pro-inflammatory mediators produced by interaction of both cell types may potentially exacerbate the development of demyelination in disease like MS, and this interaction may be potential therapeutic targets.

## Competing interests

The authors declare that they have no competing interests.

## Authors' contributions

DYK performed the majority of all experiments, the statistical analysis and wrote the initial version of manuscript, GUH performed the cell co-culture, and preparation and score in EAE model, and JYR designed all experiments, performed the data analysis and wrote the manuscript. All authors read and approved the final version of the manuscripts.

## Supplementary Material

Additional file 1 **Figure S1**. Intracellular Ca^2+ ^level, surface molecules or cytokine mRNA expression in co-cultured-astrocytes. Click here for file

Additional file 2 **Figure S2**. CD40 siRNA transfection or time courses for activities of Rho family GTPases, PKC isoforms or MAP kinases in co-cultured-astrocytes. Click here for file

Additional file 3 **Figure S3**. Time courses for activities of transcription factors or effects of inhibitors on activities of PKC isoforms or MAP kinases in co-cultured-astrocytes. Click here for file

Additional file 4 **Figure S4**. Effects of inhibitors on activities of transcription factors or expressions of cytokine mRNA in co-cultured-U87 cells. Click here for file

Additional file 5 **figure S5**. Effects of anti-TNFR1 antibody pretreatment on the co-cultured-U87 cells. Click here for file

Additional file 6 **Figure S6**. Infiltration of inflammatory cells and co-localization of TNFR1 and mast cells in EAE-induced mouse brain. Click here for file
