# Supplementary material for: Signal pathways in astrocytes activated by cross-talk between of astrocytes and mast cells through CD40-CD40L
Source: J Neuroinflammation. 2011 Mar 16;8:25. doi: 10.1186/1742-2094-8-25 (PMC3068960; doi:10.1186/1742-2094-8-25)
Supplement: Additional file 1 — Figure S1. Intracellular Ca2+ level, surface molecules or cytokine mRNA expression in co-cultured-astrocytes. [file 1742-2094-8-25-S1.PDF]

Additional file 1, Figure S1

A

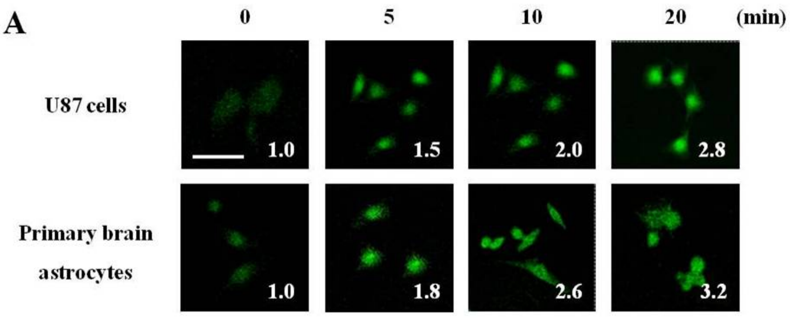

B

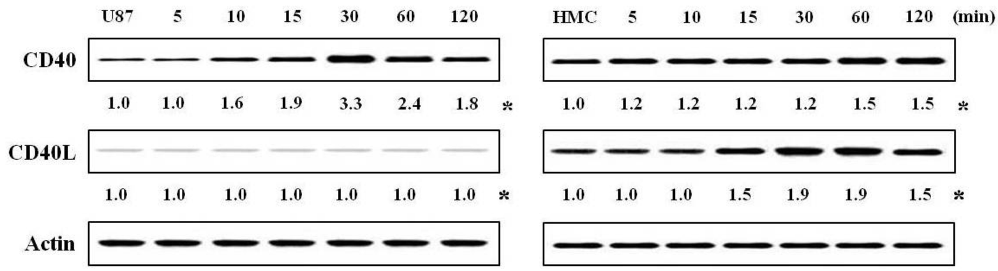

C

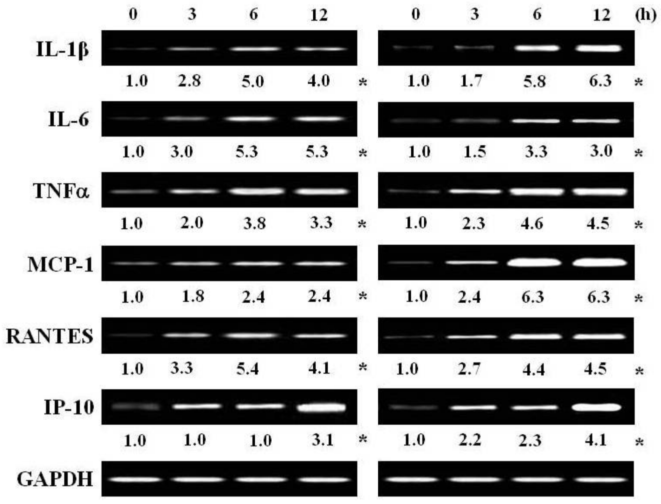

Additional file 1, Figure S1. Intracellular Ca^{2+} level, surface molecules or cytokine mRNA expression in co-cultured-astrocytes. Astrocytes (3 × 10^6 cells) and mast cells (1 × 10^6 cells) were co-cultured as ratio 3:1. Fluro-3 AM (5.0 μM) was added to co-cultured-U87 cells or -primary astrocytes and incubated for 30 min. The $[Ca^{2+}]_i$ level was analyzed in U87 cells co-cultured with HMC-1 cells (co-cultured-U87 cells) or in primary astrocytes co-cultured with BMMCs (co-cultured-primary astrocytes) using confocal laser scanning microscopy. CD40 and CD40L or each cytokine mRNA expression was determined in protein extracts and nuclear extracts using western blot and RT-PCR analysis, respectively as described in "Methods". (**A**) Intensity (shown as the numbers) of fluorescence by confocal microscopy. (**B**) Expression of CD40 or CD40L in co-cultured-U87 cells (left panel) and -HMC-1 cells (right panel). (**C**) Expressions of cytokines mRNA in co-cultured-U87 cells (left panel) and -primary astrocytes (right panel), respectively. *, Numbers below bands are mean values obtained from four independent experiments (n = 4) as the ratio band density of each group versus those of control and actin or GAPDH using densitometry analysis. Expressions of CD40 and CD40L molecules were previously shown in "J. Immunol. 2010; 185: 273-283.", but these bands are the data yielded by re-experiments. Bar in zero min indicates 100 μm.
