# Supplementary material for: Signal pathways in astrocytes activated by cross-talk between of astrocytes and mast cells through CD40-CD40L
Source: J Neuroinflammation. 2011 Mar 16;8:25. doi: 10.1186/1742-2094-8-25 (PMC3068960; doi:10.1186/1742-2094-8-25)
Supplement: Additional file 2 — Figure S2. CD40 siRNA transfection or time courses for activities of Rho family GTPases, PKC isoforms or MAP kinases in co-cultured-astrocytes. [file 1742-2094-8-25-S2.PDF]

Additional file 2, Figure S2

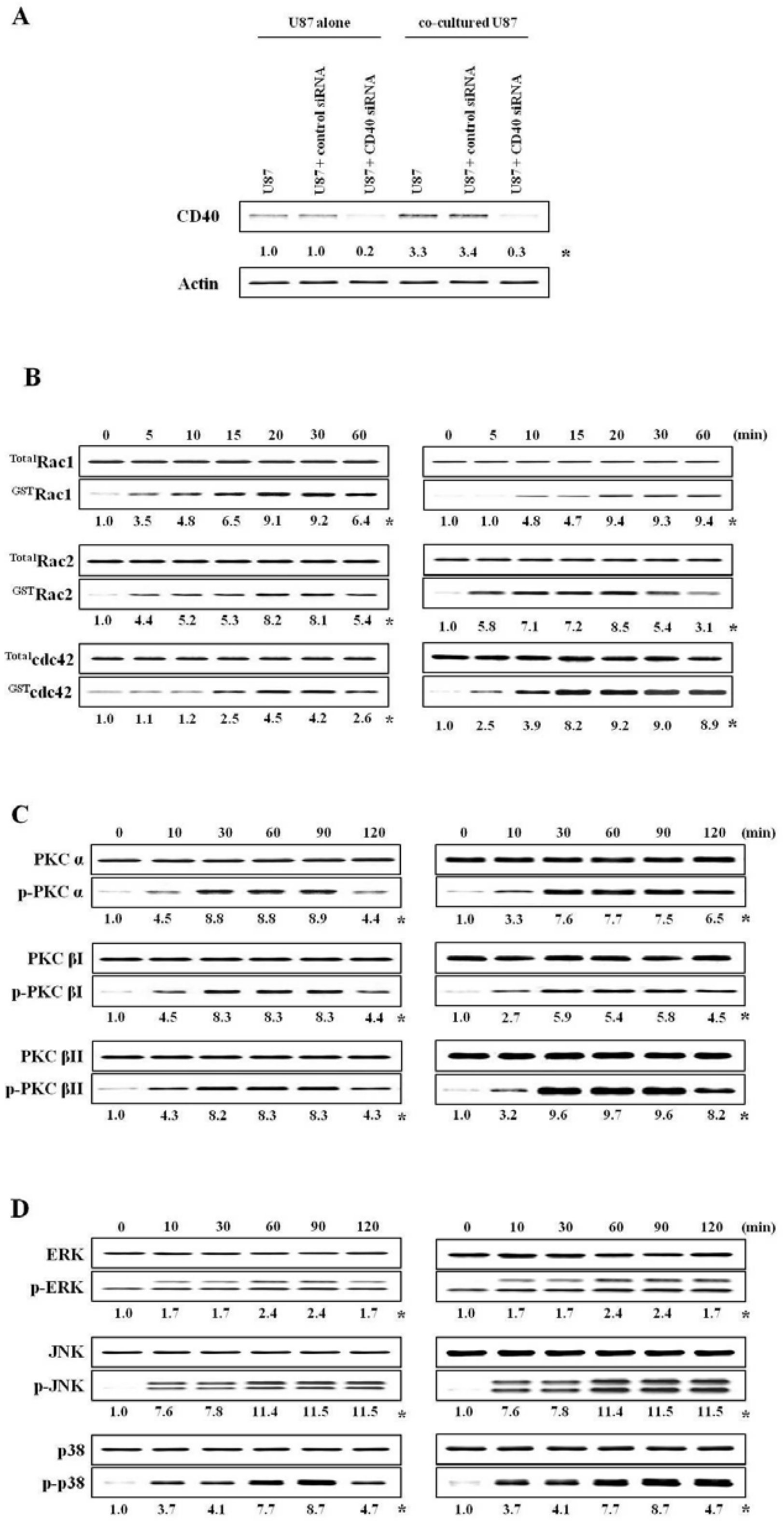

Additional file 2, Figure S2. CD40 siRNA transfection or time courses for activities of Rho family GTPases, PKC isoforms or MAP kinases in co-cultured-astrocytes. Experimental details in co-culture were indicated in additional file 1, Figure Sl. CD40 siRNA was transfected along with commercial materials as described in "Methods". The activities of Rho family GTPases, PKC isoforms and MAP kinases were determined in protein extracts using GST pull-down assay and western blot, respectively. Left and right panel, co-cultured-U87 cells and -primary astrocytes, respectively. (**A**) CD40 expression after CD40 siRNA transfection in U87 cells. (**B**) Time courses for each Rho family GTPase activity. (**C**) Time courses for each PKC isoform activity. (**D**) Time courses for each MAP kinase activity. *, Numbers below bands are mean values obtained from four independent experiments (n = 4) as the ratio band density of each group versus that of each total protein using densitometry analysis.
