# Supplementary material for: Signal pathways in astrocytes activated by cross-talk between of astrocytes and mast cells through CD40-CD40L
Source: J Neuroinflammation. 2011 Mar 16;8:25. doi: 10.1186/1742-2094-8-25 (PMC3068960; doi:10.1186/1742-2094-8-25)
Supplement: Additional file 3 — Figure S3. Time courses for activities of transcription factors or effects of inhibitors on activities of PKC isoforms or MAP kinases in co-cultured-astrocytes. [file 1742-2094-8-25-S3.PDF]

Additional file 3, Figure S3

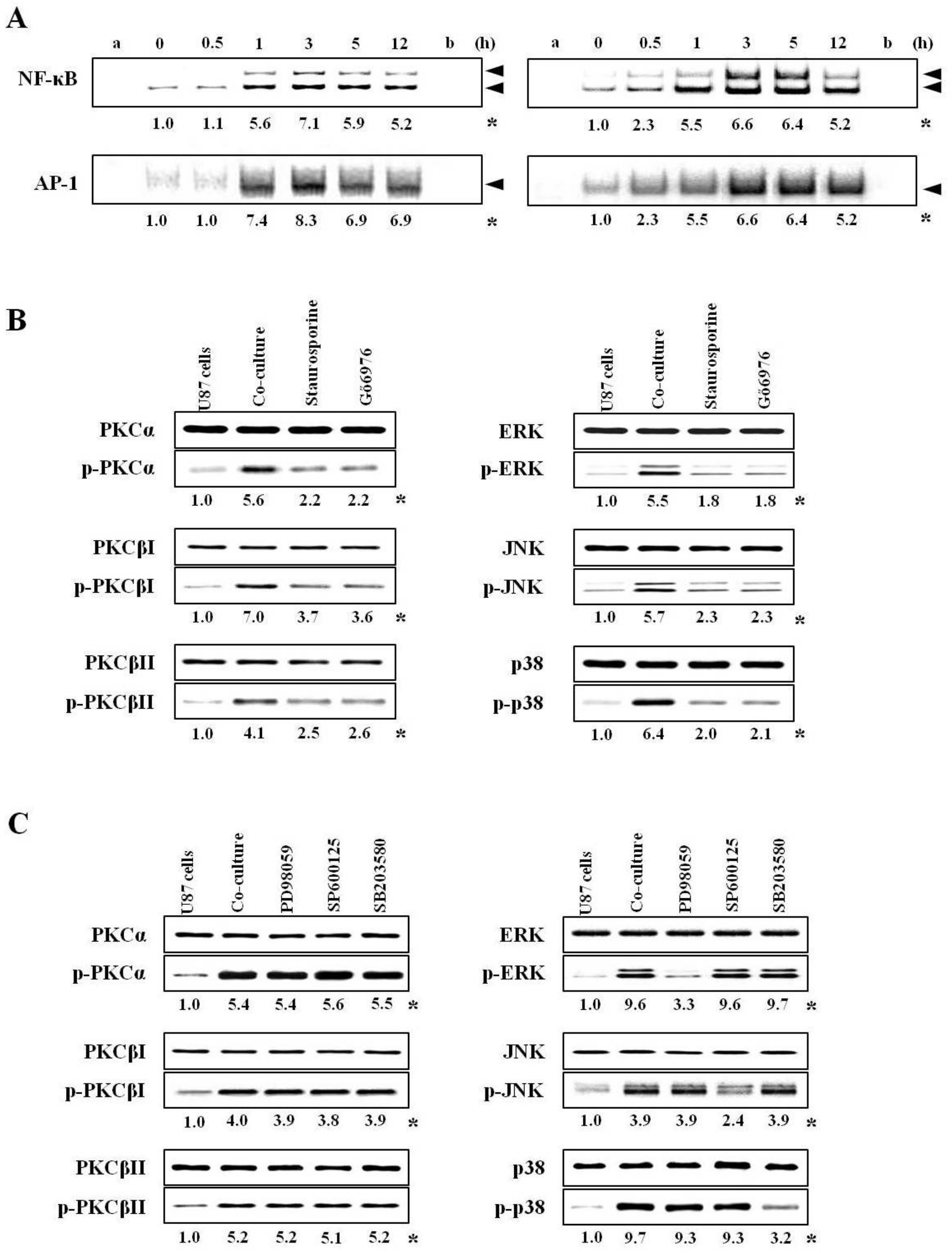

Additional file 3, Figure S3. Time courses for activities of transcription factors or effects of inhibitors on activities of PKC isoforms or MAP kinases in co-cultured-astrocytes. Experimental details in co-culture were indicated in additional file 1, Figure Sl. Transcription factors, PKCs or MAP kinases were determined in nuclear extracts and protein extracts using EMSA and western blot, respectively. PKC inhibitors (5 nM staurosporine and Gö6976) or MAP kinase inhibitors (50 μM PD98059 for ERK, 10 μM SP600125 for JNK and 10μM SB203580 for p38) were pretreated 10 min before co-culture. Left and right panel, co-cultured-U87 cells and -primary astrocytes, respectively. (**A**) Time courses for each transcription factor activity. (**B**) Activities of PKC isoforms and MAP kinases by PKC inhibitors. (**C**) Activities of PKC isoforms and MAP kinases by MAP kinases inhibitors. a, negative control; b, competition assay; U87 cells, U87 cell culture alone; Co-culture, U87 cells co-cultured with HMC-1 cells. *, Numbers below bands are mean values obtained from four independent experiments (n = 4) as the ratio of each band density of PKCs or MAP kinases versus those of control and total proteins using densitometry analysis.
