# Supplementary material for: Signal pathways in astrocytes activated by cross-talk between of astrocytes and mast cells through CD40-CD40L
Source: J Neuroinflammation. 2011 Mar 16;8:25. doi: 10.1186/1742-2094-8-25 (PMC3068960; doi:10.1186/1742-2094-8-25)
Supplement: Additional file 4 — Figure S4. Effects of inhibitors on activities of transcription factors or expressions of cytokine mRNA in co-cultured-U87 cells. [file 1742-2094-8-25-S4.PDF]

Additional file 4, Figure S4

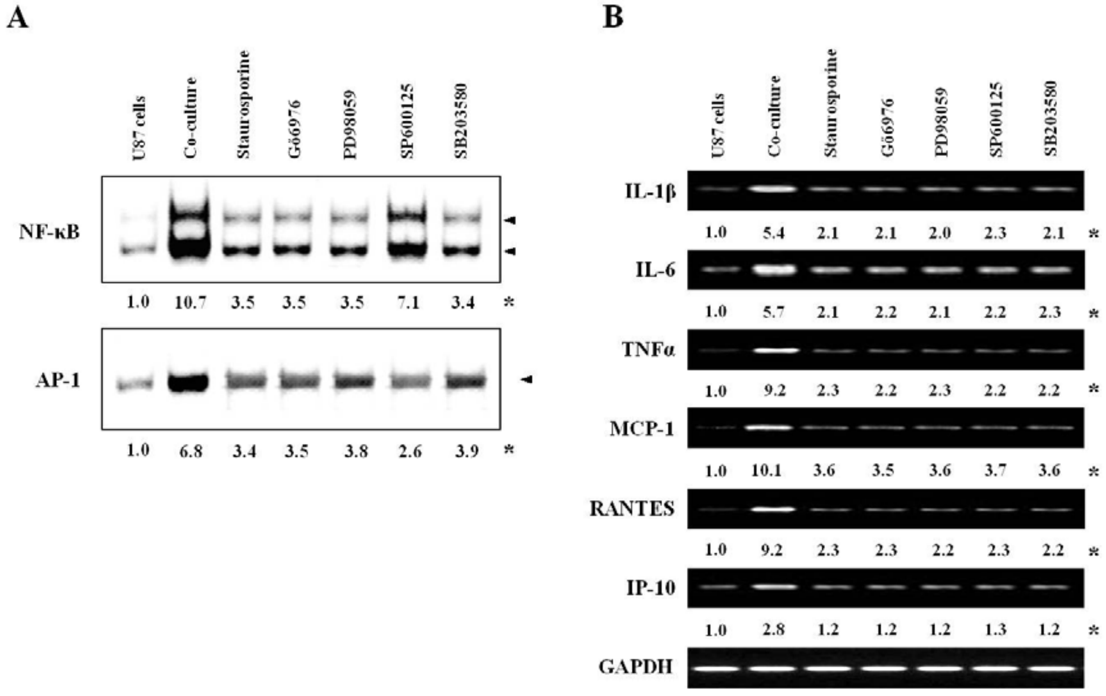

Additional file 4, Figure S4. Effects of inhibitors on activities of transcription factors or expressions of cytokines in co-cultured-U87 cells. Experimental details in co-culture and inhibitor pretreatment were indicated in additional file 1, Figure S1 and S3. The anti-CD40 antibody (300 ng/mL), Jak inhibitor (10 μM AG490) or 8-oxo-dG (300 μg/mL) was pretreated in astrocytes 1 h, 5 and 10 min, respectively, before co-culture, and CD40 siRNA was performed, as described in "Methods". (**A**) Activities of transcription factors by PKC or MAP kinase inhibitors. (**B**) Expressions of cytokine mRNA by PKC or MAP kinases inhibitors. U87 cells, U87 cell culture alone; Co-culture, co-cultured-U87 cells. *, Numbers below bands are mean values obtained from

four independent experiments (n = 4) as the ratio of each band density of PKCs, MAP kinases or STAT1 versus those of control and total proteins using densitometry analysis.
