# Supplementary material for: Signal pathways in astrocytes activated by cross-talk between of astrocytes and mast cells through CD40-CD40L
Source: J Neuroinflammation. 2011 Mar 16;8:25. doi: 10.1186/1742-2094-8-25 (PMC3068960; doi:10.1186/1742-2094-8-25)
Supplement: Additional file 5 — figure S5. Effects of anti-TNFR1 antibody pretreatment on the co-cultured-U87 cells. [file 1742-2094-8-25-S5.PDF]

Additional file 5, Figure S5

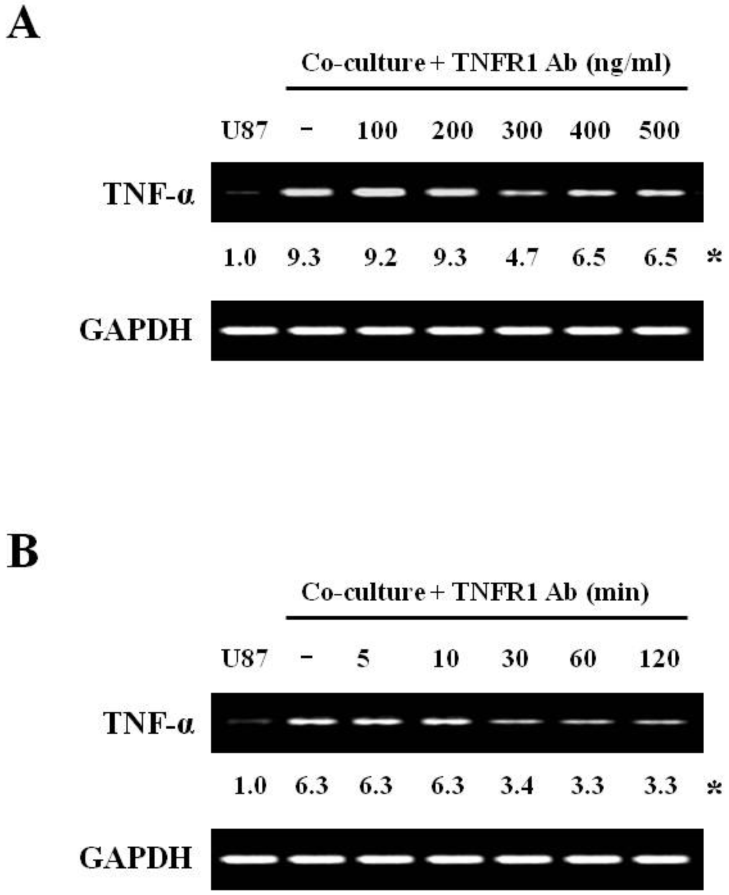

Additional file 5, Figure S5. Effects of anti-TNFR1 antibody pretreatment on the co-cultured-U87 cells. Experimental details in co-culture were indicated in additional file 1, Figure Sl. Anti-TNFR1 antibody was pretreated by a variety of dose indicated. Expression of TNF-α mRNA was determined in nuclear extracts using RT-PCR. The optimal concentration (**A**) and time (**B**) for anti-TNFR1 antibody pretreatment were 300 ng/mL and 30 min. *, Numbers below bands are mean values obtained from four

independent experiments (n = 4) as the ratio of each band density of TNF-α mRNA versus those of control and GAPDH using densitometry analysis.
