# Supplementary material for: Signal pathways in astrocytes activated by cross-talk between of astrocytes and mast cells through CD40-CD40L
Source: J Neuroinflammation. 2011 Mar 16;8:25. doi: 10.1186/1742-2094-8-25 (PMC3068960; doi:10.1186/1742-2094-8-25)
Supplement: Additional file 6 — Figure S6. Infiltration of inflammatory cells and co-localization of TNFR1 and mast cells in EAE-induced mouse brain. [file 1742-2094-8-25-S6.PDF]

Additional file 6, Figure S6

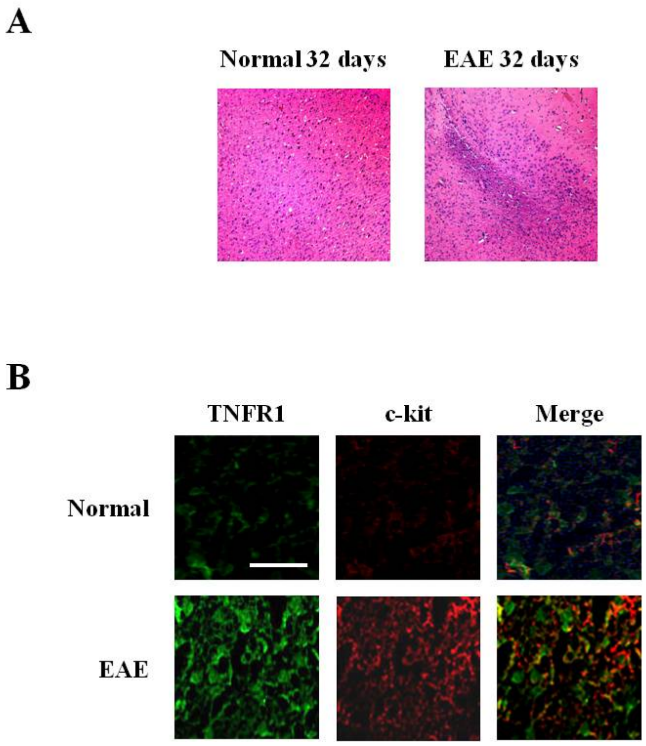

Additional file 6, Figure S6. Infiltration of inflammatory cells and co-localization of TNFR1 and mast cells in EAE-induced mouse brain tissues. EAE mouse model was immunized with MOG and CFA as described in "Methods". After mice were sacrificed at day 32 (EAE score, 3.8 ± 0.21), brains were removed and preserved in 10% neutral buffered formalin. (A) Inflammatory cells recruited in the EAE brain tissues using hematoxilin and eosin (H&E) staining. (B) Co-localization (yellow) of mast cells and TNFR1 was indicated by double staining for mast cell surface mark c-kit (red) and TNFR1 (green). Numbers of total experimental animals were four (n = 4). Bar in zero minutes indicates 100 μm
